# The First Case of Adult-Onset Periodic Fever, Aphthous Stomatitis, Pharyngitis, and Adenitis Syndrome with Splenomegaly in Iran

**DOI:** 10.22088/cjim.10.2.231

**Published:** 2019

**Authors:** Shahla Abolghasemi, Hesam Adin Atashi, Elahe Paydar-Tali, Maedeh Olya, Hamid Zaferani-Arani

**Affiliations:** 1 Department of Rheumatology, Tehran Medical Sciences Branch, Islamic Azad University, Tehran, Iran; 2 Young Researchers and Elite Club, Tehran Medical Sciences Branch, Islamic Azad University, Tehran, Iran

**Keywords:** Adult, PFAPA, Splenomegaly, Iran

## Abstract

**Background:**

Periodic fever, aphthous stomatitis, pharyngitis, and adenitis syndrome (PFAPA) is an auto-immune based disease known as a syndrome for pediatrics which typically occurs in children ≤ 5 years of age, but in 2008, for the first time, one adult case of this disease was reported.

**Case Presentation:**

Our case, a 19 year-old young man who is the first adult-onset PFAPA patient in Iran and was accompanied by splenomegaly. Since March 2017, the patient suffered from periodic febrile attacks (39-40 °C). During these fever attacks, the patient had many aphthous ulcers in his mouth, swollen glands in his neck and sore in the back of the throat. The patient was admitted to a hospital in Tehran during a severe fever attack due to weakness, lethargy, high-temperature and slight abdominal pain in the left upper quadrant (LUQ) area. Abdominal sonography was done and spleen size was larger than normal and was determined to be 32×140 mm (splenomegaly). All data were collected from a reliable governmental laboratory in Tehran.

**Conclusion:**

Following the rejection of the causes of other diseases, according to the patient's symptoms, the diagnosis of adult-onset PFAPA was given to the patient and the patient was cured with one dose of long-acting Betamethasone ampoule 1ml intravenous at the onset of fever attacks. The disease has remitted after injection of this medicine at the onset of each attack rapidly after about 2-3 hours. Also, Montelukast has been given to the patient and we saw his febrile attack intervals increased.

Periodic fever, aphthous stomatitis, pharyngitis, and adenitis syndrome (PFAPA) is an auto-immune based disease described as sudden attacks of high fever on a regular basis (periodic fever) (1) with sores in mouth (aphthous stomatitis) (2), sore throat (pharyngitis) and swollen glands (adenitis). The cause of PFAPA has not been known yet. Its reasons have not been understood yet; the body’s inflammation system becomes active, leading to the symptoms of PFAPA. Although the cause of this syndrome has been difficult to identify, the effects on lifestyle can be notable. Previous studies have shown antibiotics do not alter symptoms from this condition, but corticosteroid therapy has shown to reduce fever quickly (3). The frequency of PFAPA has not been known and may be the most common recurrent fever (auto inflammatory) that does not come from an infection. Both males and females and all ethnic groups can develop PFAPA. In the past, for a long time this disease known as a syndrome for pediatrics typically occurs in children 5 years of age and under, but in 2008 for the first time one adult case of this disease was reported (4). 

Our case is the first adult onset PFAPA patient in Iran accompanied by splenomegaly

## Case presentation

The patient is a 19 year-old boy with normal body mass index (BMI) and normal blood pressure. He was born and has lived in Tehran city (Iran) and for first the time since March 2017, he suffered from periodic febrile attacks (39-40 °C). During these fever attacks, the patient had many aphthous ulcers in his mouth, swollen glands in his neck and sore in the back of the throat. The fever periods in this patient lasted about 7 days, and after the disease went down, the next attack occurred about 45 days later, the patient also had a feeling of weakness, severe fatigue in the limbs, and a slight abdominal pain in LUQ area during the time of the attacks.

For first time in July 2017, the patient was admitted to the hospital during a severe fever attack due to weakness, lethargy, and high temperature. Doctors noticed the high fever (39.8 °C), severe aphthous month ulcers inflammation in throat, and swollen lymph nodes on the neck during physical examination. Furthermore, after examining the patient's abdomen, doctors found splenomegaly in this patient.

**Figure 1:**
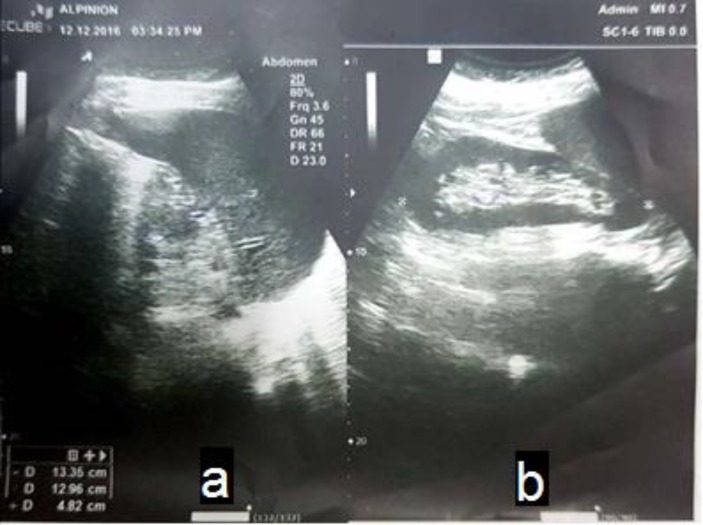
a) The abdomen sonography shows increased echo in spleen parenchyma. b) The abdomen sonography shows the spleen size enlargement

After these examinations, during the hospitalization in July 2017, doctors requested some laboratory tests to determine the cause of the disease. These tests include: urine culture (UC), blood culture (BC), stool exam (SE), stool culture (SC), human immunodeficiency virus antibody (HIV Ab), hepatitis B antigen (HBS Ag), hepatitis C virus antibody (HCV Ab), rheumatoid factor (RF), erythrocyte sedimentation rate (ESR), peripheral blood smear (PBS), malaria, borrelia, fluorescent antinuclear antibody (FANA), anti-cyclic citrullinated peptide (anti CCP), which the results of all these tests were negative except ESR (numerical value of ESR was 31), and the patient was given an abdominal ultrasound examination to determine the size of the spleen. It was larger than normal, and was determined to be 32 × 140 mm (splenomegaly) (figure 1 A, B).

Following the rejection of the causes of other diseases, according to the patient's symptoms, the diagnosis of adult onset PFAPA was given to the patient and the patient was cured by slow intravenous injection of one dose of long-acting betamethasone ampoule (1 ml) at the onset of the fever attacks. Disease remission after injection of this medicine at the onset of each attack rapidly after about 2-3 hours, and all symptoms included oral ulcers, inflammation of the throat and swollen neck lymph nodes on the neck were completely resolved. In October 2017, the patient was treated with tab Montelukast sodium 10 mg once a day. After taking this medicine, the febrile attack periods occurred once every 55-65 days, and the duration of remission increased. The laboratory tests were taken in July 2017 during the patients fever attack when patient was admitted in the hospital.

## Discussion

In this study, we report the first adult-onset case of PFAPA in Iran that had signs and symptoms since was 19 years old. This case is the first patient with adult-onset PFAPA in Iran. There are a few studies on adult-onset PFAPA in the world. The adult-onset of PFAPA is a new discovery, since it has been specified in 2008 (4, 5). This is the reason that this disease used to be thought that its onset only belongs to children before 5 year-old (6-8) or about 18 months (9-11). In the past, the prevalence of this syndrome is unknown so far (5). Our case has been presented, with all the signs and symptoms similar to what Marshall et al. has reported on PFAPA syndrome in 1987 (12). Our patient had periodic fever, aphthous stomatitis, pharyngitis, and cervical adenitis (6, 13). 

There are some minor symptoms like vomiting, abdominal pain, rash, nausea, headache, weakness, arthralgia, and etc. that were seen in the adult-onset of PFAPA syndrome (8) but in our case, he had only fatigue, malaise, headache, and abdominal pain from minor symptoms during the attacks. We cannot see any signs and symptoms between each attack though. The patients with PFAPA syndrome usually show febrile attacks occurring every 2 to 12 weeks (mean=4.5 weeks) but in our patient, it recurred every 6 to 7 weeks (8, 9). This fever reached the temperatures of 40-41 °C, while our case reached 39-40 °C. Also, our case had a significant splenomegaly (32×140 mm). Whereas, in previous studies, adult-onset PFAPA syndrome has not been stated while we ruled out other causes and diseases related to it. We should consider that many studies need confirmation of these 2 mentioned symptoms etc. In this study, the patient has been ruled out of his other all differential diagnoses (DDxs) including familial mediterranean fever (FMF), behcet’s disease, acquired immune deficiency syndrome (HIV/AIDS), cyclic neutropenia, tumor necrosis factor receptors-associated periodic syndrome (TRAPS), hyperglobulinemia D syndrome (HIDS), juvenile rheumatoid arthritis, autosomal dominant hereditary periodic fever syndrome (HPF), and cryopyrin-associated periodic syndromes (CAPS) (12, 14). They have ruled up 2 adult-onset PFAPA syndrome case report studies in Japan recently (5, 15). 

There is no study on adult onset patient of PFAPA syndrome in Iran though. In our patient, bacterial, fungal, and viral tests were done and all of them were negative. (5, 17) Our case has no leucocytosis and neutropenia. To distinguish adult-onset PFAPA from other DDxs, 5 points should be attended; 1. present illness 2. signs & symptoms 3. family history 4. genetic tests and 5. response to corticosteroids. From these 5 key points, in our study we only have not done genetic tests. With regard to genetic factors, autosomal recessive and dominant inheritance patterns were seen in FMF, TRAPS & CAPS patients, respectively. Whereas, there is no specific inheritance pattern found for PFAPA syndrome yet. Likewise, mutation in MEFV, TNFR1, and NLRP1 can be seen in FMF, TRAPS, and CAPS. If genetic tests cannot be done like our case, we can give the patient a single dose of prednisolone (60 mg) (4) and fast response (a few hours) is visible whereas rapid response to corticosteroids in other auto inflammatory diseases is seen very rarely.

Nowadays, the treatment of PFAPA is divided into 2 methods; 1. medical (nonsurgical) 2. surgical. In medical treatment, patients should take oral corticosteroids, cimetidine, and Montelukast. In surgical methods, we can often suggest tonsillectomy (18).

There are no definite treatments for PFAPA, because its pathogeneses have not been discovered clearly so far. But some previous studies have suggested empirical treatments such as prednisolone (1-2 mg/kg), betamethasone (0.1-0.2 mg/kg), or dexamethasone (3-6 mg/daily). These types of corticosteroids have been suggested to be taken at the duration of febrile attack and attack episodes. They are so useful for getting rid attack of signs and symptoms, it cannot prevent future relapse though (4, 8, 19).

Some previous studies have mentioned that the main theory and causes of PFAPA disease can be explained by T-helpr1 related immune cytokines, overexpression of Interleukin-1, and interferon-affected genes (5, 20, 21). There are so many studies about the consumption and benefits of cimetidine which proposed a 400-800 mg/day dosage based on previous studies (5, 16) although we have not given our patient at all. A study reported that giving Montelukast to PFAPA patients increased the febrile attack intervals (21). Our patient was prescribed this medicine and had maximum response.

The strong points of this study are: 1. reporting a rare disease in the world (PFAPA) that had a splenomegaly, 2. Diagnosis and timely treatment of the disease, 3. Increase the effect of treatment and interval between periods of recurrence of disease with the use of Montelukast; and the weak point is the side effects of Montelukast, which include an increased risk of respiratory infections and gastrointestinal disturbances, of which, given the PFAPA's irreversible side effects appear to be beneficial and the side effects of these drugs can be overlooked.

About the limitations, the cooperation of the patient and his family was difficult for us since they have hardly been responding to our questions and providing us medical records and lab tests.

In Conclusion, We have identified the first case of PFAPA syndrome in Iran that has been adult onset. Adult-onset PFAPA syndrome is a rare type. For the diagnosis of adult-onset PFAPA, the physicians should rule out other DDxs that we did in our study. Our case had splenomegaly in addition to Marshall et al.’s symptoms. We ruled out all DDxs with splenomegaly. Our case had all major Marshall et al’s. symptoms. Furthermore, he had fatigue, malaise, headache, and abdominal pain in minor criteria during the attack episodes. We gave Montelukast to our patient-case and we saw a good effect. With this prescription his remission duration has increased to 55-65 days. Nonetheless, other studies are needed to be investigated in terms of its effectiveness. 
